# Geometry-dependent band shift and dielectric modification of nanoporous Si nanowires

**DOI:** 10.1038/s41598-017-14647-8

**Published:** 2017-10-31

**Authors:** W. B. Yu, G. Ouyang

**Affiliations:** Key Laboratory of Low-Dimensional Quantum Structures and Quantum Control of Ministry of Education, Synergetic Innovation Center for Quantum Effects and Applications (SICQEA), Hunan Normal University, Changsha, 410081 China

## Abstract

In order to obtain a detailed understanding of the modulation of electronic properties in nanoporous Si (np-Si) nanowires with containing ordered, nanometer-sized cylindrical pores, we propose a theoretical method to clarify the band shift and associated with the dielectric modification determined by the geometrical parameters, including nanowire diameter, pore size, pore spacing and porosity, in terms of size-dependent surface energy and atomic-bond-relaxation correlation mechanism. Our results reveal that the self-equilibrium strain induced by the atoms located at inner and outer surfaces with high ratio of under-coordinated atoms as well as elastic interaction among pores in np-Si nanowires play the dominant role in the bandgap shift and dielectric depression. The tunable electronic properties of np-Si nanowires with negative curvature make them attractive for nanoelectronic and optoelectronic devices.

## Introduction

Nanoporous Si (np-Si) nanowires with negative curvature is one of the most important semiconductors that are used in many applications related to sensors, photovoltaic cells and dielectric materials^1^. Therefore, the modulation of electronic and optoelectronic properties of np-Si has attract intense research interest due to their widely application in nanoscale physics and silicon-based devices^2–5^. For example, compared with silicon-based insulators, np-Si as substrate isolation material in Si-integrated RF devices shows a lot of significant advantages^6–9^, including elimination of RC signal delay, reduction of the power consumption and wire cross talk. These advantages are realized as a result of that controlled porosity could reduce the effective permittivity.

In fact, curvature is the amount that depicts the deviation for the surface of a geometric object from being a flat plan. For the case of nanoscaled systems, two kinds of nanostructures have been taken into account: those having nanostructures with positive curvature (e.g., solid nanocrystals and nanowires) and those having nanostructures with negative curvature (e.g., nanocavities and shell-core configurations, etc.). It is generally accepted that the porous materials with negative curvature can be considered as a diphase composite, in which air is dispersed in the matrices.

Currently, some classical effective medium approximation (EMA) models that involve the volume fractions of the components have been proposed to estimate the effective dielectric constant of the porous materials, such as Maxwell–Garnett^10^, Vegard’s Law^11^, Lichtenecker^12^, Bruggeman^13^, Looyenga^14^ and Bergman^15^ methods, etc^16,17^. In general, the origin of dielectric depression is often attributed to the opening of the gap which should lower the polarizability^16^ and a local reduction of the polarizability in the surface region^17,18^. Recently, there are other accurate solutions for the dielectric constant of porous Si. Liu *et al*.^19^ put forward a theoretical method for predicting dielectric constant of through-hole and closed-pore materials by introducing structure factor. Pan *et al*.^20,21^ and Rahmoun *et al*.^22^ developed a parallel and serial model for the porosity dependence of dielectrics for np-Si. Experimentally, Adamyan and co-workers^23^ elaborated a method for determination of dielectric constant by means of analysing capacitance measurements of np-Si. Sarafis *et al*.^7,8^ integrated coplanar waveguide transmission lines on top of porous Si and measured their S-parameters to obtain dielectric parameters. Nevertheless, in spite of a number of attempts had been devoted to the understanding of dielectric properties of np-Si, the physical mechanism on the geometry-dependent band shift and dielectric modification in np-Si nanowires has not been clear. For example, the geometrical factors, including pore size, spacing and porosity, as well as the elastic interaction among nanopores, dependence of dielectric properties have not yet been taken into account from the perspective of atomistic origin.

In general, with decreasing size of materials, the ratio of surface-to-volume (SVR) will increase, and then the surface state will greatly affect the physical and chemical properties. For the case of np-Si, surface and size effects often become dominant with increasing porosity. Moreover, the loss of atom coordination numbers (CNs) near a free surface results in a corresponding redistribution of electronic charge, which alters the binding situation^24–27^. In np-Si structures, when either the pore size or the pore number increases, or the diameter of nanowire decreases, SVR will increase, and then the surface energy will greatly affect the surface state and associated with the related properties^28^. Importantly, the elastic interaction among nanopores in a specimen plays the crucial role in the related mechanical and electronic properties^29^. Therefore, it is of the utmost importance to explore the geometrical relationships in such np-Si structures for their optimal design toward enabling a broad range of technological applications.

The purpose of this study is to establish the theoretical relationship between the electronic properties, including band shift and dielectric modification, of np-Si nanowires and the geometrical parameters. Therefore, we conduct a comprehensive investigation of the joint effect of pore size, spacing and porosity as well as the elastic interaction among pores on the band shift and dielectric change of np-Si nanowires based on size-dependent surface energy and atomic-bond-relaxation correlation mechanism. We derive the analytical relationships for the dependence of the electronic properties of np-Si nanowires on various geometrical parameters. Our results reveal that the bandgap of np-Si shows a pronounced blueshift and dielectric suppression as comparable to those of the solid nanowires and the bulk counterparts.

**Figure 1:**
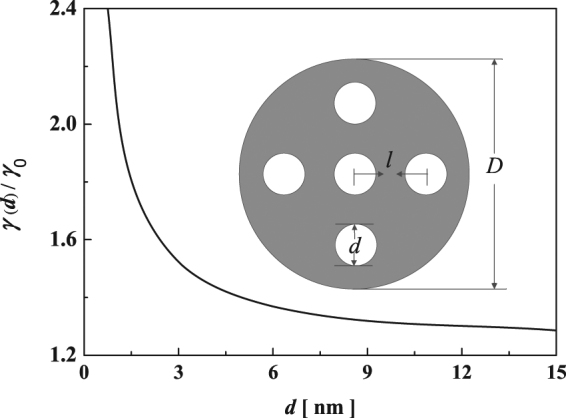
Pore size dependence of inner surface energy. The inset is a schematic illustration of np-Si nanowire.

We constructed np-Si nanowires by containing ordered, nanometer-sized cylindrical pores in regular periodic arrangements in Si nanowires, as depicted in the inset of Fig. 1. Physically, the termination of the lattice periodicity in the surface normal direction of np-Si will lead to a relaxation of CNs both in inner and outer surfaces in comparison with the bulk counterparts. It is generally known that the dangling bond of under-coordinated atoms will be shorter and stronger than that of the bulk interior. Moreover, the self-equilibrium strain induced by CN imperfection will take place spontaneously. According to size-dependent surface energy^23,28^, the lattice strain (${{\rm{\varepsilon }}}_{{\rm{\Delta }}}$) in the self-equilibrium state can be obtained by optimizing the total energy of np-Si nanowires. Thus, we have

11 $${{\rm{\varepsilon }}}_{{\rm{\Delta }}}=\{\begin{array}{c}{\varepsilon }_{i}={\varepsilon }_{i}[{\gamma }_{i}(d)]\\ {\varepsilon }_{o}={\varepsilon }_{o}[{\gamma }_{o}(D)]\end{array}$$

where the subscripts $i$ and $o$, respectively, denote the inner and outer surfaces of np-Si nanowires, $d$ and $D$ are the diameters of the pore and that of the entire nanowires, ${\varepsilon }_{{\xi }}(\xi ={i},{o})$ and $\gamma$ represent the corresponding elastic strain and surface energy density.

In addition, the total strain energy of a nanosolid with the volume ${V}_{0}$ and area ${S}_{0}$ can be expressed as: $U=\frac{{V}_{0}}{2}C{}_{\mu \nu k\lambda }\varepsilon _{\mu \nu }\varepsilon {}_{k\lambda }+{\int }_{{S}_{0}}{\gamma }_{\alpha \beta }{t}_{\alpha \mu }{t}_{\beta \nu }{\varepsilon }_{\mu \nu }d{S}_{0}$. Thus, the strain energy as accessional energy of per surface atom corresponding to ${{\rm{\varepsilon }}}_{{\rm{\Delta }}}$, can be calculated explicitly. Considering the isotropic np-Si with core-shell configuration, we can derive the single bond energy in inner and outer surfaces, i,e.,

1.2 $${E}_{js}(s=i,o)={\rm{\Delta }}{E}_{js}+{E}_{b}$$

with

1.3 $${\rm{\Delta }}{E}_{js}=u/{z}_{js}$$

here $u=\frac{{C}_{\mu \nu \kappa \lambda }{\varepsilon }_{s}^{2}/2}{\rho {N}_{A}/{v}_{0}}+\gamma 2{\varepsilon }_{s}{S}_{s}/N$,^26^ $j$ denotes the $j$ *th* atomic layer, which may be counted up to two from the outer most atomic layer to the center of the solid both in inner and outer surfaces, ${\rm{\Delta }}{E}_{js}$ and $u$ represent the single bond energy enhancement in the specific $j$ th atomic layer and the increment of cohesive energy per atom, ${E}_{b}$ and ${z}_{js}$ denote, respectively, the single bond energy of bulk atom and the effective CNs of the $j$ th layer atom, ${C}_{\mu \nu \kappa \lambda }$, $\rho$, ${N}_{A}$, and ${v}_{0}$ are the tensors of second-order elastic constants, occupies volume of silicon atoms in lattice matrix, Avogadro constant, and atomic volume, ${S}_{s}$ and $N$ are the surface area of a unit cell before relaxation and the number atoms in a unit cell. Also, the SVR $(\tau )$ for np-Si nanowires with aligned circular pores is given by

1.4 $$\tau (D,d,\eta )={\tau }_{o}+{\tau }_{i}=\frac{4h}{1-\eta }({c}_{out}/D+{c}_{in}\eta /d)$$

with $$\eta =\frac{K{d}^{2}}{{D}^{2}}$$,

where ${\tau }_{i}$ ${\rm{and}}$ ${\tau }_{o}$, respectively, are the ratio between the volume of the inner surfaces, the volume of the outmost surface shell and the volume of the lattice matrix, $h$, $\eta$ and $K$ are the bond length in interior bulk, porosity and number of pores.

Furthermore, the strain induced by CN imperfection in inner and outer surfaces of np-Si nanowires would lead to the enhancement of binding energy in the relaxed region. Thus, the cohesive energy can be expressed as follows^30^

1.5 $$\langle {E}_{COH}(D,d,\eta )\rangle =\sum _{j}{n}_{o}{z}_{jo}{E}_{jo}^{s}+K\sum _{j}{n}_{i}{z}_{ji}{E}_{ji}^{s}+{z}_{b}{E}_{b}(N-\sum _{j}{n}_{o}-K\sum _{j}{n}_{i})$$

## Results

### Band shift

**Figure 2:**
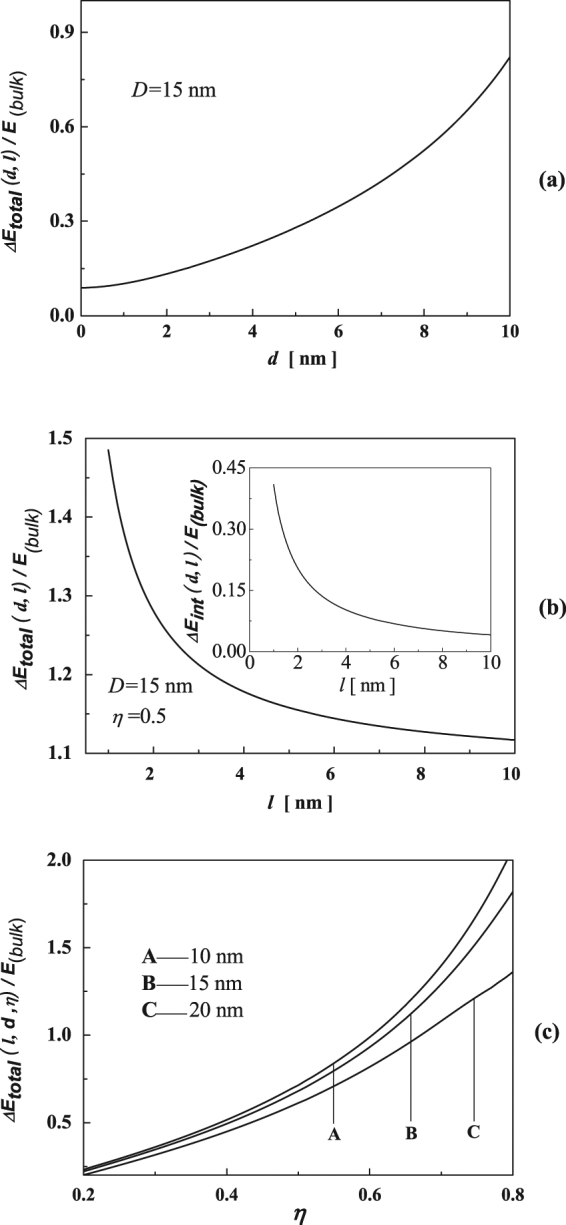
Pore size (**a**), pore spacing (**b**) dependence of relative change of total energy in np-Si with outer diameter of 15 nm and porosity-dependent relative change of total energy in np-Si with outer diameter of 10, 15, 20 nm (**c**). The inset in (**b**) shows the relationship between the relative change of elastic interaction energy and pore spacing.

As depicted in the inset of Fig. 1, $l$ is the center-to-center distance between neighboring pores. The necessary parameters are listed in Table 1. On the basis of the established model mentioned above, we first calculate the inner surface energy of nanopores in np-Si, and the results are shown in Fig. 1. Clearly, the inner surface energy increases with decreasing pore size. Interestingly, when *d* > 6 nm, the surface energy of nanopores in np-Si smoothly approaches to that of the bulk. The origin can be attributed to the changes of elastic energy and binding energy. Also, the impact of pore size, pore spacing and porosity on the relative change of total energy is shown in Figs. 2a, 2b and 2c, respectively. It should be noted that the relative change of total energy decreases with the pore size reducing. More interestingly, when *d* > 10 nm, the relative change of total energy increases dramatically as the pore size increases. In Fig. 2b, the increase of pore spacing would spark a sharp reduction in the relative change of total energy. The inset shows that the relative change of elastic interaction energy increases dramatically as the pore spacing decreases. Figure 2c shows the porosity-dependent relative change of total energy in three types of np-Si nanowires with different outer diameter of 10, 15 and 20 nm. Similarly, the change trend in three types of np-Si nanowires increases with increasing porosity. Also, the variation in quantity of np-Si nanowires with 10 nm becomes the largest among those of the other cases, which infers that the surface effect has a key role in the nanoscale.

**Table 1:** Input parameters for calculations, $h$, $a$, ${d}_{0}$, $\bar{\alpha }$, ${\gamma }_{0}$, ${E}_{g}^{B}$, $G$, $n$ and $\varepsilon$ are the bond length, lattice constant, atomic diameter, average spring constant of every pair of atoms, the surface energy density in the plane surface, bandgap, shear’s modulus, refractive index and dielectric constant, respectively.

${\bf{h}}\,{\boldsymbol{[}}{\bf{nm}}{\boldsymbol{]}}$	${\bf{a}}\,{\boldsymbol{[}}{\bf{n}}{\bf{m}}{\boldsymbol{]}}$	${{\bf{d}}}_{{\bf{0}}}\,{\boldsymbol{[}}{\bf{n}}{\bf{m}}{\boldsymbol{]}}$	$\bar{\alpha }$ [ev/Å^2^]	${{\bf{E}}}_{{\bf{g}}}^{{\bf{B}}}\,{\boldsymbol{[}}{\bf{\text{eV}}}{\boldsymbol{]}}$	${{\boldsymbol{\gamma }}}_{{\bf{0}}}\,{\boldsymbol{[}}{\bf{J}}{\boldsymbol{/}}{{\bf{m}}}^{{\bf{2}}}{\boldsymbol{]}}$	${\bf{G}}\,{\boldsymbol{[}}{\bf{\text{GPa}}}{\boldsymbol{]}}$	${\bf{n}}$	${\boldsymbol{\varepsilon }}$
0.157^44^	0.543^44^	0.22^45^	10.1^46^	1.09^47^	1.9^48^	67^49^	3.4^50^	11.8^50^

**Figure 3:**
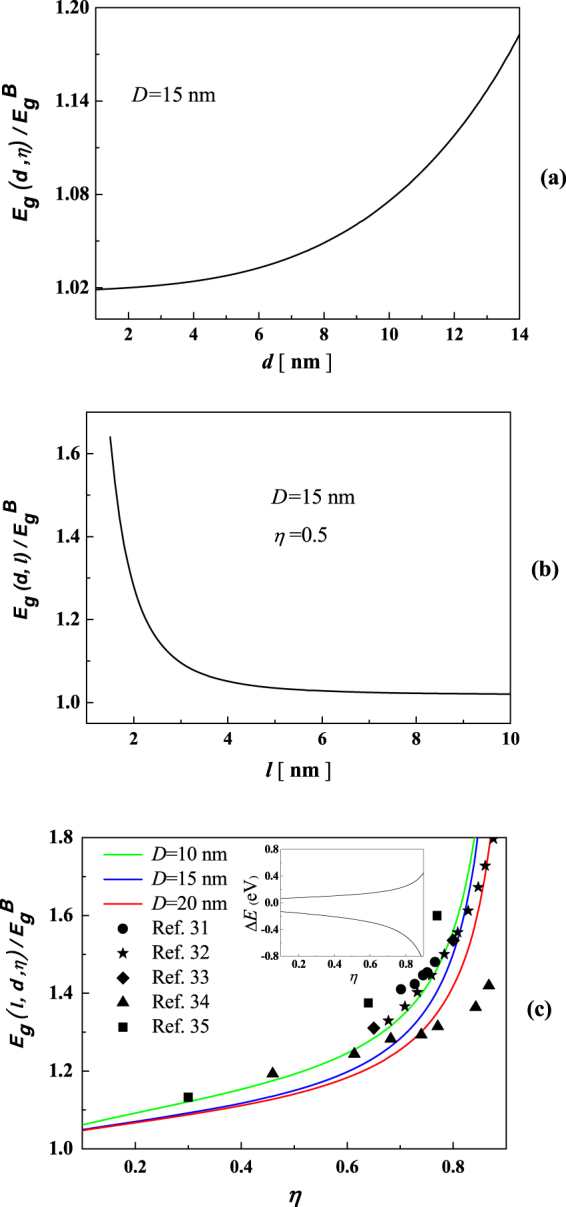
Pore size (**a**), pore spacing (**b**) dependence of bandgap shift in np-Si with outer diameter of 15 nm and porosity dependence of bandgap shift in np-Si with outer diameter of 10 (the green line), 15 (the blue line) and 20 nm (the red line) (**c**), the inset shows the shifts of conduction band and valence band, respectively.

Using Equation (2.8), we analyzed the impact of the pore size, pore spacing and porosity on the electronic properties of np-Si nanowires. As shown in Fig. 3a, the bandgap shift has a dramatic change with enlarging pore size at a fixed porosity of 0.5. Also, in Fig. 3b, the change trend of bandgap increases with expanding pore spacing. When the spacing is shorter than 2 nm, the bandgap shifts rapidly. Moreover, Fig. 3c plots the porosity-dependent band shift in three types of np-Si nanowires with different outer diameter of 10 (the green line), 15 (the blue line) and 20 nm (the red line). Note that the inset shows the shifts of conduction band and valence band. Obviously, as shown in the Fig. 3c, the bandgap shift increases gradually with increasing porosity. For example, when the porosity is 0.3, the shifts of bandgap in three types of np-Si, respectively, are 8.7%, 9.1% and 12%, while for the porosity increases to 0.7, the bandgap shifts is 25%, 28% and 33%.

### Dielectric constant modulation

**Figure 4:**
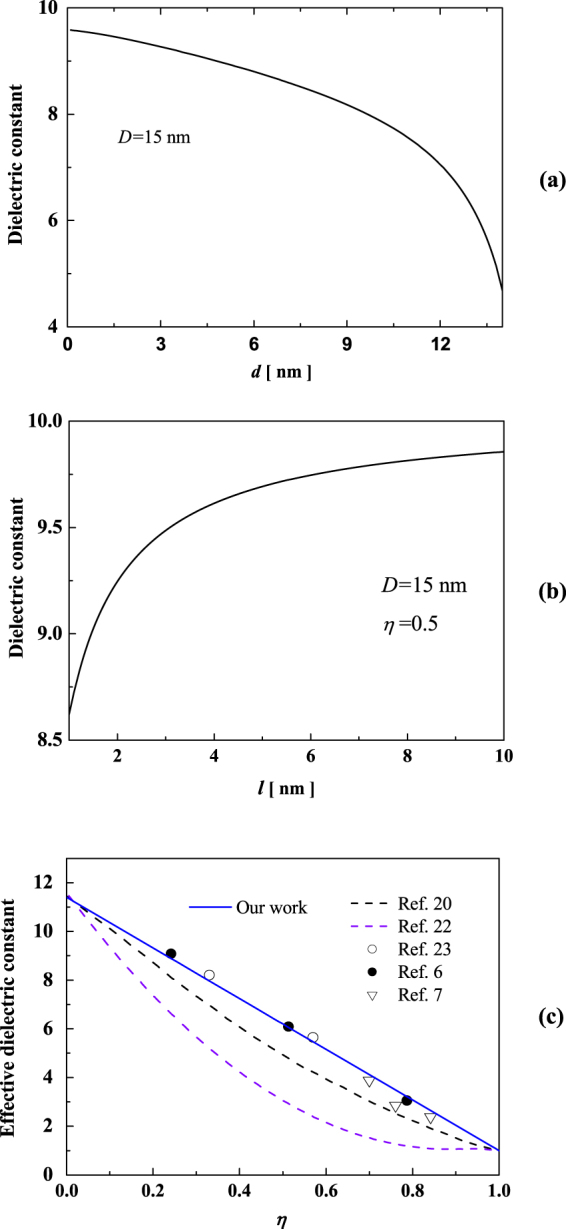
Pore size (**a**) and pore spacing (**b**) dependence of dielectric constant in np-Si with outer diameter of 15 nm, porosity dependence of effective dielectric constant in np-Si with outer diameter of 15 nm (**c**).

Figure 4 shows the pore size, pore spacing and porosity dependence of dielectric constant in np-Si nanowires. In Fig. 4a, the dielectric constant decreases with increasing pore size. Interestingly, when the pore size is larger than 10 nm, the dielectric constant decreases rapidly. Meanwhile, Fig. 4b shows the dielectric constant increases with widening pore spacing, which have not been mentioned in the current classical models. Also, the effective dielectric constant in three types of np-Si nanowires reduces as the porosity increases, as depicted in Fig. 4c.

## Discussions

In our case, we investigate the joint effect of geometrical parameters involved in pore size, pore spacing and porosity on the band shift and dielectric modulation of np-Si nanowires. Our results show that the changes of porosity, pore size or spacing would alter bandgap and further affect the dielectric properties. First, we find that the bandgap shift becomes more and more obviously with enlarging pore size. In this case, the inner surface area increases with the pore size enlarging, which leads to a lot of surface CN imperfection. Second, different pore spacing results in the change of elastic energy. As illustrated in Fig. 3b, we have analyzed the impact of the pore spacing on the electronic properties of np-Si nanowires and demonstrated the bandgap shift. This phenomenon is mainly caused by the fact that the elastic interaction among pores, which becomes stronger as the pore spacing shrinks. As a result, the lattice energies and electronic band structures would change. When the separation among pores gets larger, the elastic interaction gradually grows weaken and the band shift reduces. Third, from Fig. 3c, we find that the bandgap of np-Si nanowires expands with increasing porosity. In our case, the pore size of np-Si with small outer diameter is much smaller than that of np-Si with large outer diameter under the condition of identical porosity. Larger SVR means that the CN imperfection is more remarkable and the surface effect becomes more obvious^24–27^. It is noted that the self-equilibrium strain induced by surface effect changes the electronic properties due to different bond identities. Importantly, our predictions are exceedingly well agreement with the experimental measurements and theoretical calculations^31–35^.

Meanwhile, we clarify the related mechanism on the pore size, pore spacing and porosity dependence of dielectric constant of np-Si nanowires at the fixed outer diameter of 15 nm. In Fig. 4a, the dielectric constant increases with shrinking pore size. As shown in Fig. 4b, the dielectric constant decreases as the pore spacing narrows down. The reason is mainly from the elastic interaction among pores, which greatly becomes stronger with shrinking pore spacing. Also, in Fig. 4c, we can see that the effective dielectric constant of np-Si nanowires decreases linearly with increasing porosity. The variation trends are consistent with the experimental observations^6,7,23^. For example, Sarafis *et al*.^7^ obtained almost linear relationship between the real part of dielectric permittivity and porosity. Kim *et al*.^6^ found that the relative dielectric constant of porous Si films linearly decreased from 9 to 3 with increasing porosity from 24% to 78%. However, our results differ from other theoretical methods^20,22^. In fact, the origin of disagreement is ascribed to the fact that the surface oxidation had been considered. Previous classical EMA studies^36,37^ attributed the reduction of dielectric constant to the lower matter polarization density. Lannoo *et al*.^17^ and Delerue *et al*.^18^ suggested that the decrease of average dielectric response with decreasing size is due to the breaking of polarizable bonds at the surface, Pan and co-workers^16^ proposed that the physical mechanism on the dielectric reduction is due to the bandgap expansion instead of the breaking of polarizable bonds. In our case, the self-equilibrium strain induced by surface effect and elastic interaction among pores change the band shift and suppress the dielectric constants. Our results show that the effective dielectric constant is not only related to the porosity, but to pore size and pore spacing as well.

To summarize, we have investigated the geometry-dependent electronic properties of np-Si nanowires based on size-dependent surface energy and atomic-bond-relaxation method. An analytic model has been presented to address the band shift and associated with the dielectric constant change for np-Si nanowires from the perspective of atomistic origin. It is found that the self-equilibrium strain in np-Si nanowires, arising from CN imperfection and elastic interaction, causes the band shift and hence the dielectric depression. Our results demonstrate that the bandgap of np-Si with small outer diameter and large porosity is higher than that of np-Si with large outer diameter and small porosity. Importantly, our predictions are consistent with the available evidence. Therefore, the proposed method not only can shed light on the effects from self-equilibrium strain and elastic interaction among nanopores, but also provide an effective way of shape design on tunable electronic properties of silicon-based nanostructures and nanodevices.

## Methold

### General consideration on the strain energy induced by elastic interaction among pores

Strain induced by elastic interaction among pores, will modify chemical potential and hence changes the properties of materials. Thus, the elastic interaction energy among pores must be taken into account. The relationship between elastic interaction force ${F}_{el}$ and total elastic interaction energy ${E}_{int}$ is given by^29^

2.1 $${F}_{el}=-\frac{\partial {E}_{int}}{\partial l}$$

where $l$ is the separation among pores. In terms of Hooke’s law, the stress per unit area $\sigma$ caused by elastic interaction among pores can be expressed as

2.2 $$\sigma =\lambda \varepsilon +2G\varepsilon $$

where $G$ is the shear modulus, $\lambda =\nu E/(1+\nu )(1-2\nu )$ is the Lame constant, with $E$ the Yang’s modulus and $\nu$ the Poisson’s ratio.

In order to exactly illustrate the interaction among pores, the effect of the surface energy of each pore needs to be considered, i.e.,

2.3 $${E}_{int}=\frac{1}{2}\sum _{j=1}^{n}{\int }_{SUR}-{P}_{j}{\mu }_{j}dS$$

where the integral are taken over the surface of each pore, ${\mu }_{j}$ is the displacement in the $j$ th hole, and ${P}_{j}$ is the elastic pressure in the $j$ th $(j=1,2,\cdots n)$ pore, i.e.,

2.4 $${P}_{j}=2{\gamma }_{j}/{r}_{j}$$

where ${\gamma }_{j}$ is the inner surface energy of the $j$ th pore, ${r}_{j}$ is the radius of pore. In the light of size-dependent surface energy^38^, $\gamma$ can be composed of two parts: one is chemical and the other is structural: $\gamma ={\gamma }^{chem}+{\gamma }^{stru}$. In detail, the chemical part of the surface energy originates from the bond energy at the inner surface, while the structural part is from the elastic strain energy in the inner skin of nanopores, which can be written as:

2.5 $$\gamma (d)={\gamma }_{0}(1+4{d}_{0}/d)+\bar{\alpha }{\varepsilon }^{2}$$

In Equation (2.5), ${\gamma }_{0}$, ${d}_{0}$, $\bar{\alpha }$, and $\varepsilon$ are the values in the plane surface with zero curvature, the atomic diameter, and the average spring constant of every pair of atoms, and the deformation lattice of the inner skin. Thus, we have

2.6 $${E}_{{\rm{int}}}(l,r)=\sum _{i\ne j}\frac{\pi {r}^{3}{P}^{2}}{G}(\frac{r}{{l}_{ij}})$$

with ${l}_{ij}$ is the separation between the $i$ th and $j$ th pore, Thus, the relative change of total energy is

27 $${\rm{\Delta }}{E}_{total}(D,d,\eta ,l)/E({\rm{bulk}})=[\langle {E}_{COH}(D,d,\eta ,l)\rangle +{E}_{int}(l,r)]/N\langle z\rangle {E}_{B}-1$$

The joint effect of CN imperfection of surface atoms and pore elastic interaction contributes to a perturbation to the total Hamiltonian that determines the electronic band structure. Physically, the bandgap is stemming from the crystal potential and can be calculated by the first Fourier coefficient of the crystal potential energy, which is proportional to the mean energy per bond $\langle {E}_{0}\rangle$,i.e., ${E}_{g}\propto \langle {E}_{0}\rangle ={E}_{total}/(N\langle z\rangle )$, where $N$ and $\langle z\rangle$ are, respectively, the number of atoms and the average CNs.

Therefore, the ratio between the bandgap of np-Si nanowires and that of the bulk counterparts can be obtained

2.8 $$\frac{{E}_{g}(D,d,l,\eta )}{{E}_{g}^{B}}=\frac{{z}_{b}}{ < z > }\{\begin{array}{c}\sum _{j}{\tau }_{jo}[{z}_{jo,b}\frac{{E}_{jo}^{s}}{{E}_{b}}-1]\\ +\sum _{j}{\tau }_{ji}[{z}_{ji,b}\frac{{E}_{ji}^{s}}{{E}_{b}}-1]+1\end{array}\}+\sum _{i\ne j}\frac{\pi {r}^{3}{P}^{2}}{G}(\frac{r}{{l}_{ij}})/N\langle z\rangle $$

with $${z}_{jo,b}={z}_{jo}/{z}_{b}$$, $${z}_{ji,b}={z}_{ji}/{z}_{b}$$ and $$\langle z\rangle =\sum _{j}{\tau }_{jo}({z}_{jo}-{z}_{b})+\sum _{j}{\tau }_{ji}({z}_{ji}-{z}_{b})+{z}_{b}$$.

According to the theory of solid state, the dielectric constant of a semiconductor results from electrons polarization. Also, the relationship between the absorption coefficient $\alpha$ and the energy of incident waves $h\omega$ satisfies^39^

2.9 $$\alpha ={A}^{\ast }{(h\omega -{E}_{g})}^{\eta }$$

where $h\omega$ and ${A}^{\ast }$ are the incoming photon energy and a material constant, $\eta$ is a parameter that represents the type of transition (for direct transition $\eta =0.5$, whereas for indirect transition $\eta =2$)^40,41^.

In addition, the imaginary part of the complex dielectric constant ${\varepsilon }_{2}$, which is responsible for the energy loss of the incident waves by electron polarization, describes the electromagnetic wave absorption. The relation between the imaginary part of the dielectric function and the absorption coefficient $\alpha$ can be related as ${\varepsilon }_{2}=\frac{\alpha nc}{\omega }$, where *n* is the refractive index, and $c$ is the speed of light. Notably, the Kramers–Kronig relation correlates the real part ${\varepsilon }_{1}$ to the imaginary part ${\varepsilon }_{2}$ of the dielectric constant^42^:

2.10 $${\varepsilon }_{1}-1=\frac{2}{\pi }{\int }_{{\omega }_{0}}^{\infty }\frac{{\varepsilon }_{2}}{\omega }d\omega =\frac{h{A}^{\ast }}{\sqrt{{E}_{g}}}$$

where $$\,\,{\omega }_{0}={E}_{g}/h$$, $${A}^{\ast }=Anc$$.

Therefore, combining the relations mentioned above, the dielectric constant of np-Si nanowires can be calculated by

2.11 $$\varepsilon (D,d,l,\eta )=\frac{{\varepsilon }_{B}(\infty )-1}{\sqrt{{E}_{g}(D,d,l,\eta )/{E}_{g}^{b}}}+1$$

Furthermore, the effective dielectric constant of np-Si nanowires is predicted by volume weighted effective medium theory^43^, namely

2.12 $${\varepsilon }_{eff}=[{\varepsilon }_{0}-\varepsilon (D,d,l,\eta )]\eta +\varepsilon (D,d,l,\eta )$$

where ${\varepsilon }_{0}$ is the dielectric constant of air.
